# A Secure and Portable Multi-Sensor Module for Distributed Air Pollution Monitoring

**DOI:** 10.3390/s20020403

**Published:** 2020-01-10

**Authors:** Gyorgy Kolumban-Antal, Vladko Lasak, Razvan Bogdan, Bogdan Groza

**Affiliations:** Faculty of Automatics and Computers, Politehnica University of Timisoara, Timisoara 300223, Romania; kolumbanantal@yahoo.com (G.K.-A.); vladko.lasak@student.upt.ro (V.L.); bogdan.groza@aut.upt.ro (B.G.)

**Keywords:** pollution measurement, sensor networks, security, authentication, group signatures

## Abstract

Air quality in urban environments has become a central issue of our present society as it affects the health and lives of the population all over the world. The first step in mitigating negative effects is proper measurement of the pollution level. This work presents a portable air pollution measurement system, built from off-the-shelf devices, that is designed to assure user privacy and data authenticity. Data is collected from sensor modules that can be hand carried or installed on vehicles, possibly leading to a vehicular sensor network that may cover a larger area. The main challenge is to provide authenticity for the sensor data while also ensuring user privacy. The proposed system assures authenticity and non-repudiation for the collected data by using group signatures and a blockchain-like structure for secure storage. We use regular key-exchange protocols based on elliptic curve cryptography in order to securely bootstrap a session key, then we benefit from secure tunneling to export data from sensors to the remote server. Post-update tampering is prevented by the use of a blockchain-like structure on the data server. We carry experiments both to determine the computational requirements of the procedures, as well as to measure indicators of air quality on nearby areas.

## 1. Introduction and Motivation

Ambient air pollution is one of the most widespread environmental hazards in urban settlements as it affects the population’s health all over the world. Air quality of urban agglomerations is a key agenda item today at the planetary level. This was proven by the call for action of world leaders in Katowice, Poland, during the COP24 UN Climate Change Summit that took place in December 2018. According to a World Health Organization study in 2015, air pollution costs European economies more than 1.5 trillion US dollars each year, in diseases and deaths, nearly equivalent to 10% of the Gross Domestic Product (GDP) of the entire European Union [1]. Increasingly, researchers are finding that air pollution chemical compositions coming from increasing urbanization, the cooling and heating systems of buildings, along with traffic jams are a significant variable in health impacts, causing chronic cardiovascular and respiratory diseases and lung cancer. However, supporting datasets are still limited [2].

Authorities in different countries are trying to, not only monitor air quality parameters, but also develop measures that can counteract the effects of different pollutants. Current approaches for monitoring air pollution use expensive, stationary equipment, which limits the spatial resolutions of the measurements, making them relevant for macro-scale air quality mapping [3]. This type of system relies on the principle that a single node covers a large urban area and will retain the location of each measurement. The core of such solutions are high precision gas sensors that come with increased deployment and maintenance costs.

The low-cost middle precision sensors are an alternative to this type of sensor, being built mainly for indoor air quality measurements, but having the possibility to successfully be used for outdoor operations. Therefore the current trend in pollution monitoring is to replace massive, costly, and seldom reference sensing stations with mobile and micro-scale sensing solutions, which could offer accurate information in a real-time fashion [4].

In this work, we pursue the design of a low-cost mobile urban air pollution monitoring system based on mobile sensor nodes and middle precision sensors. The solution forms a vehicular sensor network (VSN), the used sensor nodes being placed on vehicles moving around the city. The most convenient approach has been demonstrated [3] to be the public transportation fleet, as its vehicles cover a large geographical area in a short time. This paper presents the experimental set-up and results in the town of Timisoara, situated in the West part of Romania.

Giving the fact that the data is transmitted wirelessly by the sensors of the network, in order to assure the security and privacy of the VSN, we rely on cryptographic security. Using existing cryptographic protocol suites, e.g., Secure Sockets Layer/Transport Layer Security (SSL/TLS), is not an option since these are just tunneling protocols and cannot assure specific goals such as the authenticity of the data received from the sensor modules or anonymity for the users. Our solution tries to address both security and privacy concerns by using standardized cryptographic blocks, that are known to be secure, in a protocol that is suitable for our needs. In principle, we use a modified version of the Station-to-Station (STS) protocol in order to exchange a secret session key. To assure a user’s anonymity within a group, we rely on group signatures by which the signer cannot be traced inside the group. Nonetheless, we store sensor data in a block-chain structure that achieves immutability and which makes it impossible to alter data that was already committed. More details on the security design will be given in a forthcoming section.

Our work is structured as follows. Section 1.1 discusses the state-of-the-art regarding VSN, pollution monitoring and security solutions. Section 2 is focused on the design of the proposed system and gives details on the security design. Experimental results are presented in Section 3 and further developments and conclusions are in Section 4.

### 1.1. Related Work

In order to measure the emissions of pollutants, a spectrum of systems has been developed around the world. Different air monitoring solutions that use hardwired or wireless connected sensors have been presented in the scientific literature. To increase measurement accuracy, one of the adopted techniques is implementing neural networks in order to predict temperature and humidity based on gas concentration values [5]. In [6] a product is described that builds on hand-held mobile devices, which cover different parts of the city, but the dataset is limited since the monitoring was manual. Based on the gathered data, different predictions are offered for the covered areas.

A low-cost (around 150 USD) mobile pollution sensing device called M-pod [7,8] has been developed at the University of Michigan and University of Colorado Boulder, and has been tested in indoor environments. It supports the detection of different air pollutants, like CO, ${\mathrm{CO}}_{2}$, ${\mathrm{NO}}_{x}$, ozone, and volatile organic compounds (VOCs), but also can measure temperature, humidity, and light. Using a Li-ion battery with a capacity of 6000 mAh, the total life-cycle of the system is around 12 h. Based on the M-pod device, the paper proposed sensor placement techniques in order to minimize the drift error of individual sensors.

Another indoor air quality measurement is presented in [9]. This device combined multiple sensors to form a multi-sensor sensor node and used 1 transmitter for sending the data. The final cost for the entire product is around 150 USD. It does not need GPS and mobile internet and does not use a VOC sensor like other existing approaches. The presented experiments were mainly related to fire detection in the buildings, although the 109 USD ${\mathrm{CO}}_{2}$ sensor was also used for indoor air pollution measurements.

Taking into account the advantage of mobility, vehicular sensor networks have been proposed as monitoring solution by different authors. Such networks are formed by mobile sensor nodes that are carried by vehicles. Dedicated middlewares were proposed in [10] for VSN deployment, and are able to spread sensed data summaries toward vehicles from the near vicinity. In [11], VSN were combined with Vehicular Ad-hoc NETworks (VANET) in order to reduce the costs associated with communication. In [3] the urban air quality measurement is accomplished using a VANET. The bus fleet of Palermo, in Italy, was used to accommodate the sensor nodes. The data regarding air quality is gathered during bus trips, but this solution is not providing real time pollution monitoring due to the fact that the data is uploaded to a central server when the bus arrives to each station.

The problem of obtaining efficient dissemination by using cloud to vehicle communication is studied in [12]. Based on a greedy algorithm, a vehicle route-based data prefetching scheme is offered, and the success probability of data dissemination is maximized. The work in [13] presents a solution for optimal deployment costs in vehicular sensor networks based on wireless sensor networks. In [14] the air quality index is used to measure monitoring accuracy, while the amount of sampling data is a parameter for communication cost. The offered solution is called efficient data gathering and estimation (EDGE) and is based on dynamic grid partitions having the variation of pollutant concentration as input. The algorithm computes a probabilistic reporting to prevent potential network congestions.

Event-monitoring and data-gathering frameworks and quality of service (QoS) optimizations are presented in [15,16,17]. In [18] a VSN air pollution monitoring network based on low-cost gas sensors is presented. This approach, however, does not account for security issues involved in the deployment of such solutions.

The problem of security in wireless sensor networks (WSNs) has been well addressed in the literature. One of the central issues is how to securely exchange a session key that can be later used to perform encryption and message authentication. Pair-wise key sharing and pre-distribution of secret keys has been extensively studied for WSN, e.g., [19]. To facilitate an interactive key exchange between sensor nodes, the use of asymmetric cryptography is the only alternative. Elliptic curve cryptography has been previously deployed for sensor networks in [20]. The advantage coming from the use of elliptic curves stems from the small key sizes that are more convenient for WSN.

When developing applications and services in VSN, one of the key factors to be offered to drivers and passengers is user authentication, while still preserving privacy for the user. Such solutions may call for more expensive cryptographic operations, e.g., bilinear pairings [21], which are a building block for group and identity-based signatures. Group signatures, which stay at the core of our proposal, have been also explored by numerous works, e.g., [22,23,24,25,26]. It is commonly recognized that such signatures are beneficial in preserving the anonymity of group members. There are a number of differences between the aforementioned approaches. For example, Wu et al. [25] avoids the use of a bilinear pairing operation to lower the computational costs. For the same reasons, Wasef and Shen [23,24] use batch verification, while their proposal still makes use of bilinear pairings. The work in [26] seems largely based on symmetric cryptography.

However, one of the main limitations of these works is that they do not provide concrete experimental results regarding the performance of group signatures, which are highly demanding from a computational perspective. In contrast to these, we present clear experiments on a commonly used micro-controller and use a well known group signature scheme [27], which is known to be secure. While the computational demands make group signatures somewhat unsuitable for V2V communication (which requires low latencies), they are well suited for our scenario of pollution monitoring, which does not require a fast response from sensor nodes.

Many other works are worth mentioning. The work in [28] proposes an ID-based batch signature without using bilinear pairings. This signature scheme utilizes a general one-way hash function in order to consume less computing time. This ID-based signature scheme is further applied in order to develop a conditional privacy-preserving authentication scheme. In order to guarantee message authenticity sent between vehicles and roadside units, Asaar et al. [29] proposes an identity-based message authentication scheme using proxy vehicles (ID-MAP), without bilinear pairings. The certificateless public key cryptography is used in the authentication scheme of [30]. This scheme uses elliptic curve multiplication instead of bilinear pairings due to reduced computational costs. The case of authentication for the Internet of Vehicles is addressed in [31] by using a double pseudonym method in order to hide the real identity of vehicles. In [32] the trust relation between vehicles is calculated based on connectivity duration, centrality, and security level. In this way, the security level adaptation is provided as a means to improve the QoS of safety applications from VSNs.

In order to assure secure communication in VANETs, different approaches [33] have been discussed in the literature. In this context, Gayathri et al. [34] presents a pairing-free, certificateless authentication scheme that uses batch verification, while [35] introduces an identity-based fault-tolerant batch verification. In [36] the solution is based on a password-based conditional privacy preserving authentication and group-key generation protocol. The authors in [37] present a solution for solving the problem of the security bottleneck of a trusted third party existent in VANET by using identity-based cryptography and short lifetime region-based certificate. Targeting lower message delays and message loss ratios, the authors of [38] proposed a certificateless signature with message recovery. In [39], the identity-based data transmission protocol uses Lagrange interpolation for integrity protection, while [40] addressed the anonymity in VANETs by proposing an anonymous and lightweight authentication based on a smart-card protocol.

## 2. System Architecture and Protocol Design

We begin this section by discussing the components on which the system is built. Subsequently, we proceed to the design of the security protocol.

### 2.1. System and Components

**Figure 1:**
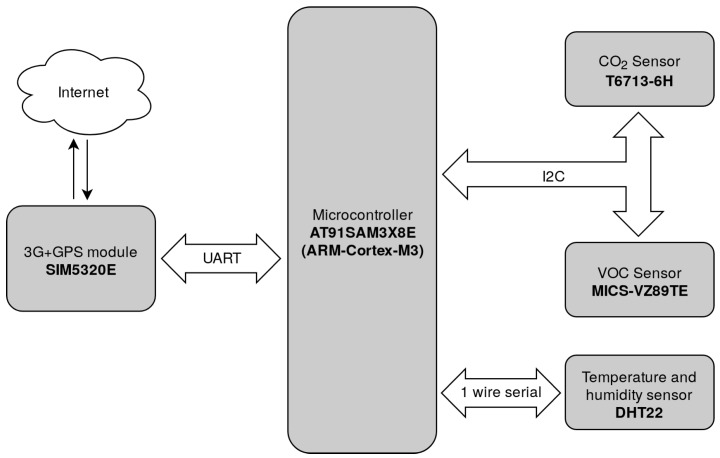
Sensor nodes system diagram.

**Figure 2:**
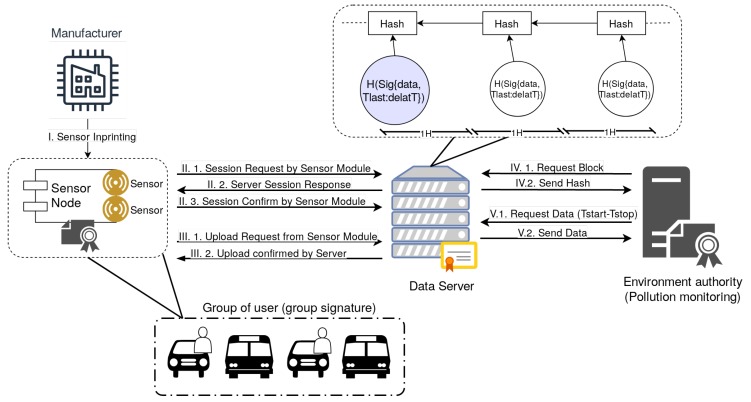
Overview of the proposed system.

The hardware architecture of the proposed sensor nodes is presented in Figure 1. The components are divided into system components (Table 1) and sensors (Table 2). The sensor node is composed of multiple sensors, a microcontroller, and a 3G/GPS module for communication and localization. Figure 2 shows only the most important components of the system, we omit power sources, antennas, and logic level converters, in order to avoid overloading the figure.

The sensor nodes are based on the AT91SAM3X8E microcontroller, using the ARM Cortex-M3 architecture. The microcontroller runs on 84 Mhz clock frequency and is equipped with 96 KB SRAM and 512 KB flash memory. The communication protocol required careful design due to these limitations of the platform.

There are two air quality sensors connected to the microcontroller through the Inter-Integrated Circuit (I2C) protocol. The first sensor is the T6713-6H ${\mathrm{CO}}_{2}$ concentration sensor. The ${\mathrm{CO}}_{2}$ sensor measures from 400 to 5000 ppm, with a precision of ±30 ppm + 3% of the reading. Currently the atmospheric ${\mathrm{CO}}_{2}$ concentration is 413 ppm [41], so the T6713-6H is not able to produce fine grained air quality data, but it is able to detect abnormal concentrations.

The second air quality sensor is the MICS-VZ89-TE that measures volatile organic compounds (VOC) concentration in the air. As the name suggests, this sensor measures the concentration of organic compounds that evaporate on room temperature. The odors sensed by the human nose are most often caused by different types of VOCs. Some VOCs have no health effect, while others cause cancer [42]. The list of VOCs considered pollutants differ from country to country, an example being presented in [43] for South Korea and Japan. In terms of VOC, MICS-VZ89-TE measures the total concentration (tVOC) in the range of 0–1000 ppb isobutylene equivalent. Although the MICS-VZ89-TE sensor does not indicate a difference between pollutant and non-pollutant VOCs, it detects above average concentrations.

Apart from the air quality sensors, there is a DHT22 temperature and humidity sensor connected to the microcontroller. The DHT22 sensor uses its specific one-wire serial protocol. The purpose of this sensor is to retain weather condition of the measurements, in order to study correlation between weather conditions and pollution data.

Both localization and Internet connectivity are provided by the SIM5320E chip. The GPS part of the chip is essential in order to retain location together with pollution measurements. The 3G part of the system provides rich functionalities but we need only the mobile internet connectivity on the sensor nodes. Mobile internet is used to create Transmission Control Protocol (TCP) connections to the data servers and the security protocol presented presented in the next section is built on top of the TCP protocol.

### 2.2. Protocol Design Goals

In a previous work [44], we started to explore the design and implementation of a VSN system to monitor air pollution. However, our system lacks privacy protection for the users and authentication for the data once it is stored. To assure these goals, the current solution is based on group signatures and bilinear pairings that opens the road for adding privacy to our scheme. Moreover, we use a blockchain-like structure on the data storage servers in order to assure immutability of the reported data. The design goals of our protocol are the following:

1. Developing a protocol stack that is computationally affordable for mid-range embedded devices, e.g., 32-bit Cortex M3 at 80 Mhz in our implementation.
2. Assuring authenticity and non-repudiation for values that are broadcasted by the sensor modules.
3. Assuring confidentiality of the traffic from the sensor modules to protect users from eavesdroppers.
4. Assuring privacy for users such that the reporting sensor module remains anonymous for the rest of the network.
5. Immutability, by which data cannot be changed later (this is to be assured by a blockchain-like structure).
6. Proof-of-concept implementation on ARM-based microcontrollers and data servers.

Figure 2 shows an overview of our system. The actors in our system are the following:

1. *Manufacturers* are responsible for releasing the multi-sensor modules (also referred as sensor nodes in our work) and imprinting them with the corresponding keys.
2. *Users* that are the physical entities to which the sensors belong. A user may posses one or multiple sensor modules, our assumption is that these are installed in mobile devices, e.g., cars or buses, but we do not exclude the possibility that they may be carried by persons as the modules are quite lightweight.
3. *Sensor modules* are the devices used for monitoring air quality parameters, e.g., VOC, ${\mathrm{CO}}_{2}$, etc., their architecure was already presented in the previous section.
4. *Data servers* are used for storing data from the sensors and mediates data visualization. We assume that these data servers are not necessarily trustworthy, a reason for which we store data in a blockchain-like structure and add the next entity for assuring immutability of the information.
5. The *environment authority* has the role of storing the blockchain-like structure and to verify the correctness of the data from the data servers at any later point.

We also assume that a *trusted key generation server* is present for distributing group keys and potentially for solving disputes when they arise, e.g., tracing a particular user. To avoid overloading the figure we omit this key generation server.

### 2.3. Proposed Protocol

**Figure 3:**
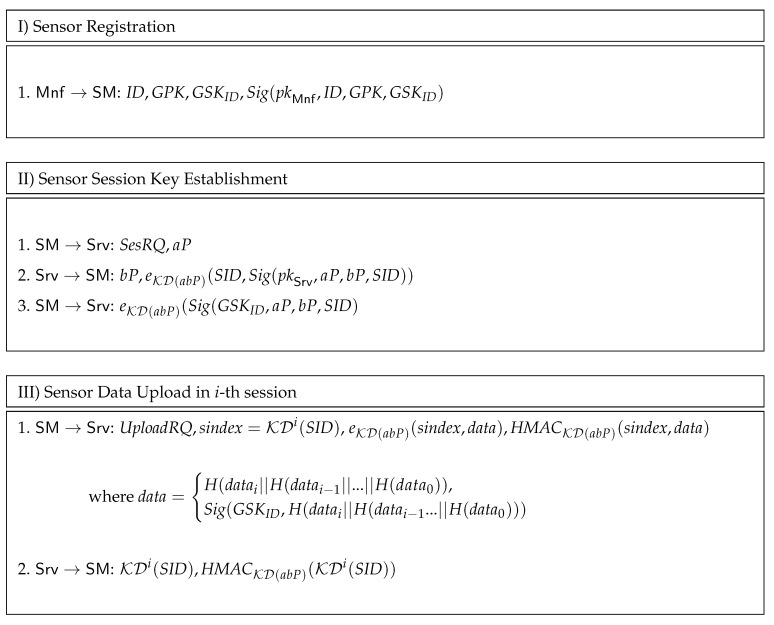
Protocols for sensor registration, session key establishment, and data upload.

Our protocol is built upon standardized cryptographic building blocks such as the Advanced Encryption Standard (AES), the Secure Hash Algorithm SHA256, the Hash-based Message Authentication Code (HMAC), and the Elliptical Curve version of the Diffie–Hellman key agreement (ECDH). For the later we use some modifications, e.g., encrypting signatures to preserve anonymity, which were proposed in subsequent enhancements of the Diffie–Hellman protocol, i.e., the Station-to-Station protocol (STS) [45]. We also rely on less standardized cryptographic blocks: We use as group signature the Boneh–Boyen–Sacham short group signature [27], which builds upon bilinear pairings. In particular, for the group signature we use the implementation made available by [46]. The rest of the cryptographic implementations come from standard cryptographic libraries that were available on our platforms and are referenced in the implementation-related section. The protocol procedures are briefly outlined in Figure 3, we now discuss each of them.

First, in step I, each sensor SM released by the manufacturer will be initialized with a unique identification number ID, the common group public-key GPK and a unique group secret key ${\mathrm{GSK}}_{\mathrm{ID}}$. The sensor also receives the signature of the manufacturer Mnf on the previous values, i.e., $\mathrm{Sig}({\mathrm{pk}}_{\mathrm{Mnf}},\mathrm{ID},\mathrm{GPK},{\mathrm{GSK}}_{\mathrm{ID}})$ so that in case when a dispute arises it can prove that these values where indeed released by the manufacturer.

In step II, the sensors has to establish a session key with the server and then upload the current environmental data. To establish the session key, the sensor SM makes a request to the server Srv denoted by a default token SesRQ followed by a Diffie–Hellman key share aP. The server Srv replies with its own Diffie–Hellman share bP followed by a message encrypted with the Diffie–Hellman session key, i.e., $e_{\mathrm{KD}(abP)}\left(\mathrm{SID},\mathrm{Sig}\left({\mathrm{pk}}_{\mathrm{Srv}},aP,bP,\mathrm{SID}\right)\right)$. To compute a symmetric encryption key we rely on a key derivation process KD, by which a symmetric key is extracted. The encrypted message contains the Diffie–Hellman shares and a session identifier SID. The role of the session identifier SID is to allow the server to determine the session key once a particular packet is received from the sensor (since connection to the server is intermittent such an identifier is necessary).

Subsequently, when the sensor SM needs to upload data, in the *i*-th session of Step III, it makes a request with UploadRQ followed by ${\mathrm{KD}}^{i}\left(\mathrm{SID}\right)$ and the encrypted data $e_{\mathrm{KD}(abP)}\left(\mathrm{data}\right)$. Here, ${\mathrm{KD}}^{i}\left(\mathrm{SID}\right)$ denotes the key derivation process repeated *i*-th times on the session identifier SID. The role of this repeated derivation is to avoid both a replay and the disclosure of the session ID SID in order to preserve anonymity in front of an adversary that eavesdrops on the connection. Once the data is uploaded in the *i*-th session, the server computes ${\mathrm{KD}}^{i+1}\left(\mathrm{SID}\right)$ and stores this on the local hash table along with the session key abP, so that when a new data upload request arrives it can easily determine the session to which it belongs. The data that is sent by the sensor is either the signed data or hash over the current and the hash of the hash of the previous values, i.e., $H({\mathrm{data}}_{i}\|H({\mathrm{data}}_{i-1}\|\ldots \|H\left(data_{0}\right))$.

Using this hash-chain structure has the role of saving computations from the regular signature that accompanies the data. In this case signing will be performed only on the last data-block, which contains hashes over all previous values. The server replies to the data upload request by sending ${\mathrm{KD}}^{i}\left(\mathrm{SID}\right)$ along with a HMAC computed on it ${\mathrm{HMAC}}_{\mathrm{KD}(abP)}\left({\mathrm{KD}}^{i}\left(\mathrm{SID}\right)\right)$. The key of the HMAC is again extracted by a key derivation process from the Diffie–Hellman session key, i.e., KD(abP).

## 3. Experimental Results

**Figure 4:**
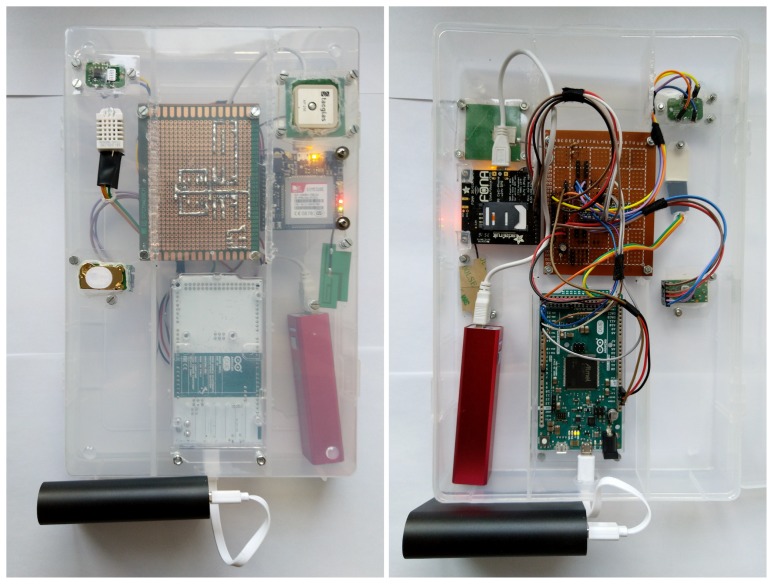
The resulting proof-of-concept multi-sensor module.

A proof of concept version of the sensor nodes and the data server have been implemented. The resulting sensor modules are depicted in Figure 4. This section begins by presenting the software architecture of the system. Later, the runtime of the cryptography algorithms, the transmission time are analyzed including optimizations. Power consumption results are also presented for the proof of concept system. Finally the resulting pollution maps are presented.

### 3.1. Implementation Details

**Figure 5:**
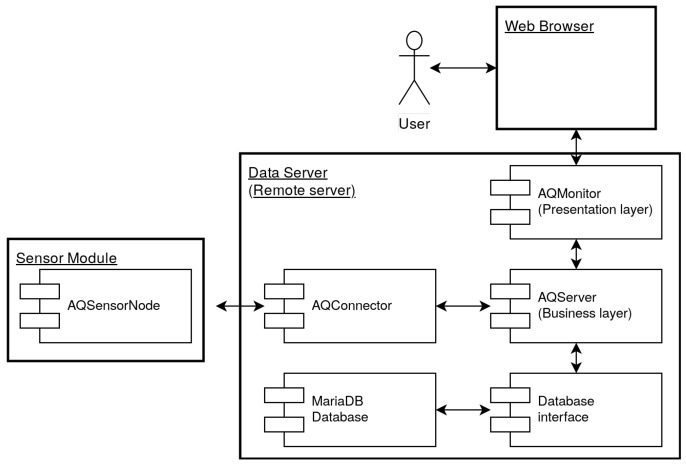
Software system architecture.

The proposed system is composed of multiple software components: the AQSensorNode, the AQConnector, the AQServer (business layer), the AQMonitor (front-end layer), and the database. These are presented in Figure 5. All software components are custom made, except for the database software (MariaDB). The AQSensorNode software is running on the sensor nodes. These sensor nodes gather data and send it to the data server. The AQConnector acts as a gateway on the data server, having role of decryption and validation of the sensor data. Valid sensor data is sent to the the AQServer. The AQServer exposes a representational state transfer application programming interface (REST API), that is used to store and retrieve sensor data from the database. Finally, the AQMonitor is responsible for the data visualization. It uses the REST API provided by the AQServer in order to obtain the necessary data for the visualization.

#### 3.1.1. AQSensorNode

The AQSensorNode software runs on the AT91SAM3X8E microcontroller (having ARM Cortex M3 architecture) and handles sensor data reading and uploading.

The AQSensorNode is interfacing multiple sensors, using multiple libraries when collecting air pollution, location, time, and weather information. This information is collected periodically and is reported back to the data server. In the current implementation, there is a delay of 10 s between the measurements.

First of all, ${\mathrm{CO}}_{2}$ and VOC levels are measured, being read through the I2C bus by the microcontroller. Next, the temperature and humidity values are obtained using the DHT22 sensor. This sensor uses its own one-wire serial protocol. In our case it is driven by the Adafruit DHT library. The measurements are not relevant without the proper location information, so GPS localization is done immediately after measurement. Both GPS localization and networking are provided by the SIM5320E chip, connected through Universal Asynchronous Receiver/Transmitter (UART) to the microcontroller. The Adafruit Fona library with some application specific extensions was used to interface with this chip.

Once the data is collected, it can be uploaded to the data server. Cryptography is required to secure the environmental data and to assure its authenticity. Several cryptographic libraries where required to implement the proposed protocol: Boneh–Boyen–Sacham short group signatures from the PairingsInC (https://github.com/IAIK/pairings_in_c) is used for client (sensor nodes) side signatures. Elliptic Curve Digital Signature Algorithm (ECDSA) and Elliptic Curve Diffie Hellman (ECDH) algorithms are provided by the micro-ECC library (https://github.com/kmackay/micro-ecc). These algorithms are required for the data server side signatures and the session key establishment. For symmetric encryption the AES part of Arduino Cryptography Library (https://github.com/rweather/arduinolibs) is being used. Hash computing is done using the SHA256 library from Brad Conte (https://github.com/B-Con/crypto-algorithms). Combining all these libraries, it is possible to perform a secure key exchange with the AQConnector and create the encrypted tunnel. The encrypted tunnel is used to transmit the sensor data, location, and timestamp, and the corresponding hash chain information. Please note, that the hash chain mentioned here is not the same as the blockchain-like structure of the data server proposed in the previous section. Short lived hash chains are used during data upload together with groups signatures in order to maintain the authenticity of the measured data. With each set of measurements, a hash value is sent. This hash value is computed on the current set of measurements concatenated with the previous hash value. (The initial hash value is selected by the sensor node and it is uploaded with the first measurement.) Hash chains are ended with a message containing a signature on the hash value. The signature assures the authenticity of all measurements included in the hash chain. This mechanism is required in order to reduce the number of signatures applied. (Signatures are require more time to compute than hash values.) When the signatures of the sensor node arrives to the data server, the current series of measurement data and hash values together with the signature can be added to a blockchain-like structure, to prevent further tampering. (The blockchain-like structure is out of the scope of this section.)

#### 3.1.2. AQConnector

The AQConnector is a service that collects the data from the sensor nodes. It participates in the session key establishment and reads data through the encrypted channel. All session keys and session ID are stored in the AQConnector, being properly updated after each transaction. Session IDs need to change, because they are visible for an eavesdropping adversary. If session key IDs would be constant, the adversary could track the sensor nodes. However, in the current system after each upload a new session ID is computed using the key derivation function. Since by eavesdropping an attacker does not have access to the key used for computing the session ID, it does not know which session IDs will follow for the same sensor nodes. In other words, the attacker can not track the sensor nodes based on the session IDs. Signature validation at the end of the hash chain is also performed by the AQConnector. In other words, the AQConnector reads the data packages and the corresponding hash values, until it receives the signature from the entire chain. When the signature arrives, and is valid, the AQConnector sends the data to the AQServer business layer. In case the signature is not valid, or it does not arrive in time, the entire chain of data is dropped. From the crytpographic perspective, the AQConnector uses the same libraries as the AQSensorNode, with architecture specific compilation flags.

Sending the valid data to the AQServer business layer is done using the REST API. In order to perform REST API calls, the AQConnector is using the Boost library (https://www.boost.org/). The REST API is further detailed under the AQServer paragraph below.

#### 3.1.3. AQMonitor, AQServer, and the Database

The core of the data server is composed of three entities: AQMonitor (implementing the user interface/front-end), AQServer (implementing the business layer/back-end), and the MariaDB database. AQMonitor is a web application, built using the Angular framework. It offers the possibility to the end user to visualize an interactive map, on which the collected data from the sensor nodes are represented. Using predefined filters, the user can select the time period and the sensor, to visualize the desired data on the map. The AQMonitor receives data using the exposed REST API from AQServer, acting as a client.

AQServer is an application based on ASP.NET Core WebAPI template, that is responsible for collecting the data from the AQConnector and providing it to the AQMonitor. The AQServer provides a REST API, acting as central point for the clients while giving the possibility to send the collected data from the sensor nodes and obtain the data stored in the database. For the received data, AQConnector uses a private endpoint exposed by AQServer. To get the environmental data the clients can use the public REST API. Using this public REST API, the environment authority is able to inspect the existing data. The data is stored in database in multiple tables using an ORM (object-relational mapper). The database used is a relational database managed by MariaDB management system. The information about sensors is stored by default in the database.

### 3.2. Computational Results

The runtime of the system has been also analyzed on multiple levels. First, the processing time required for the cryptographic algorithms have been measured. Next, the processing time of the individual steps has been analyzed. Finally, the time required for an entire report operation has been measured, including both processing time and transmission delay.

**Figure 6:**
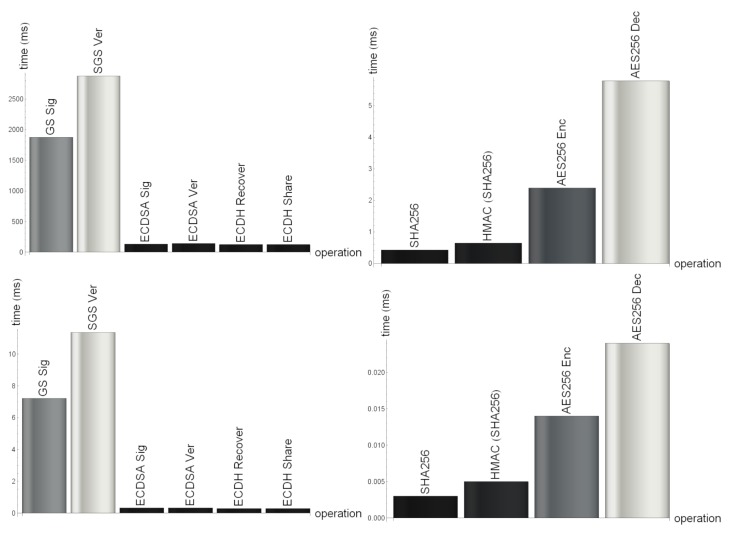
Overview of the computational results.

As expected, there are considerable differences between the runtime of the cryptographic algorithms on the Atmel AT91SAM3X8E microcontroller and the Intel Xeon E5-2676 v3 processor. The runtime results are summarized in Table 3 and graphically depicted in Figure 6.

Table 3 contains average values from 256 time measurements. Each measurement is obtained with random input data and random cryptographic keys. Key generation time is not included since key generation is not done very often. The sensor nodes relied on predefined public/private key-pairs. The input sizes are selected as the maximum input size for each cryptographic algorithm in the current implementation of our application. In the current implementation there are four sensors. By changing the number of sensors, the data package size changes and so does the input of cryptographic algorithms involved in the sensor data upload.

Time measurements on the AT91SAM3X8E microcontroller were performed with an external logic analyzer. Therefore accuracy problems of the microcontrollers clock signal have been eliminated. The Intel Xeon E5-2676 v3 processor measurements have been performed on a Linux machine, in AWS. Only CPU time has been measured, in order to reduce the impact of other tasks running on the machine. Please note, that runtime of the Elliptic Curve Cryptography (ECDSA and ECDH) algorithms is very similar, because there is a side-channel attack protection implemented in the ECC library [20] that we use.

The processing time of the cryptographic algorithms gives a proper understanding about the timing requirements of the algorithms on both sensor node and data server side. For both sides the most computationally intensive operations are the signature and verification operations with group signatures. This is, of course, expected due to the more expensive bilinear pairing. Less computational intensive are the elliptic curves based signatures, but they still have considerable processing time. During the design of the protocol, one important idea was to reduce the number of operations requiring asymmetric cryptography.

Processing time has been further analyzed, for each individual phase and each operation of the protocol. The resulting measurements are collected into Table 4.

The first operation is the session key establishment, having three phases, each corresponding to a message. Computing the first message does not require much time for the sensor node, since there is no asymmetric cryptography involved. The first and the second message computation on the data server side already includes ECDSA and ECDH algorithms, but these operations are fast on the server processor. On the sensor nodes side, the second message computation also includes elliptic curves based algorithms. Therefore, the processing time for this message is already increased. However, most of the time is spent on the third message for both parties, since only the third message includes short group signatures. In general the session key establishment is a computationally heavy operation, this is why session keys are reused for multiple data uploads.

The second operation is data upload. The time required for data upload does not include the time required for the hash chain computation. This operation only includes symmetric encryption with AES256, and HMAC computation. For both parties, this operation is not computationally demanding.

More intense computations are included for the hash chain operations. As mentioned earlier there are intermediate hash chain blocks and the final block of the hash chain. Intermediate hash chain blocks require only one hash computation; therefore, they are computationally lightweight. The final block of the hash chain requires a group signature, that is considerably more computational expensive. In order to show the differences, cases with and without group signatures have been analyzed separately.

The group signature introduces high computational overhead, so in order to reduce the average time needed for hash chain operations, the frequency of the signed messages needs to be reduced. Unfortunately, hash chains are traceable by the data server. By choosing low frequency of signed hash chain messages, the length of the hash chains increase. This means longer periods of time when, the data server can track a sensor node. From a privacy perspective hash chains should be as short as possible. Careful decision must be made about the length of the hash chain, as this implies an important compromise between processing time and anonymity. Instead of using a fixed value for the hash chain length, it is determined based on the GPS coordinates of the sensor node. When the sensor node is still, longer hash-chains are allowed. When the sensor node is in motion the length of the hash chains is reduced.

In addition to the processing time, the total time of the protocols operations has been measured in Table 5. The first two rows are giving the time for session key establishment from both the client (sensor node) and server (data server) side. The first row of the table represents the time interval required by the sensor node from the computation of the first message to the sending of the third message. Similarly the second row represents the time from the moment when the data server receives the first message, until it processes the third message. The remaining two rows are containing time required for data upload from the sensor nodes perspective, including the hash chain computation.

By subtracting the processing time from the total time of each operation, the transmission time is obtained. The transmission time includes both the request and the reply time. It can be observed, that transmission times are all between 5 and 6 s, mostly independent on the nature of the operation or on the amount of data sent. Due to the transmission times, the operations of the protocol take longer amount of time, but this is due to the networking equipment (e.g., connectivity delays) not due to the operations mandated by our protocol. Choosing other networking chip, will result in different operation times, but it is outside of the scope of this article to improve on transmission times. Several seconds are affordable due to the low frequency of reports required in practical scenarios.

The most time consuming operations are the asymmetric cryptography operations, especially the Boneh–Boyen–Sacham short group signatures. The proposed protocol is optimized by reducing the number of such operations. This section also showed that a trade-off exists between fast processing and privacy. On one hand, shorter hash chain lengths favor privacy, as the data server can track the sensor nodes for shorter periods of time. On the other hand, longer hash chains mean a smaller number of group signatures and a smaller value for the average computation time.

### 3.3. Power Consumption

**Figure 7:**
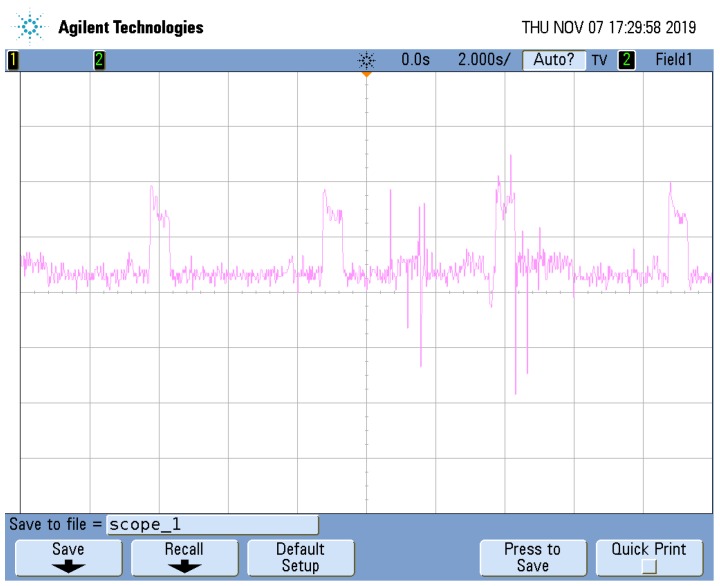
Power consumption measurements: voltage drop on an 0.56Ω resistance (horizontal scale 50 mV, vertical scale 2 s).

The sensor nodes can be powered from the vehicle’s power source, but the experimental set-up used in this paper shows that battery powered sensor nodes are also possible. In fact, the device used to carry out the experiments is also battery powered. Therefore, power consumption measurements have been performed. A 0.56Ω resistance was connected between the ground of the sensor node and the ground of the power source. The voltage drop on the resistance was measured with an oscilloscope, and plotted to Figure 7. The magenta graph expresses the actual voltage drop, the zero level is not visible in the figure, but it is 3 units above the lowest line in the figure. The vertical time scale is 2 s and the horizontal voltage scale is 50 mV. The power consumption can be divided into two components. There is the baseline component and periodic additional bursts, caused by the ${\mathrm{CO}}_{2}$ sensor. Based on the graphic, the baseline power consumption, causes a voltage drop of 177.5 mV on the resistance. Having a resistance of 0.56Ω, the current through it, is 316.96 mA. Knowing that the supply voltage is 5 V, the baseline power consumption of the system is derived as approximately 1.58 W.

In addition to the baseline, there is also a consumption burst appearing every 5 s, due to the T6713-6H ${\mathrm{CO}}_{2}$ sensor. This sensor is an optical one, and emits a light beacon. Changes in direction of the beacon are used to determine the ${\mathrm{CO}}_{2}$ concentration. The duration of the burst is 0.6 s, during which the voltage drop on the resistance increases with 65.62 mV. This means a current increase of 117.18 mA, and a power consumption increase of 0.58 W, for the period of the burst. The burst also contributes to the average power consumption with 0.070 W. Adding up both components the average consumption of the system will be 1.65 W.

A similar computation will show that the average current drain of the system is 331 mA. During the performed experiments, we used a battery of 10,400 mAh, showing that the system has approximately 31 h of autonomy.

The most significant part of the power consumption is constant and only a small overhead is introduced by the ${\mathrm{CO}}_{2}$ sensor. The resulting 1.65 W power consumption might seem much, but it includes power required by the mobile network transmissions, microcontroller based computation and the three environmental sensors. Taking into consideration all these factors, it shows that the system has more than one day of autonomy.

### 3.4. Air Pollution Monitoring

Once the entire system was implemented, air pollution and weather condition maps were created. These maps combine the measured values with the measurement locations, giving a proper perspective on the spacial distribution of various pollutants. The measured values are represented with colored dots on the maps. The lower values are colored with green, while the higher values are colored with red. It should be noted, that these maps are relative to the measured values, and do not take in consideration the health effects of various pollutants. In other words even if there are red dots on the map, it does not mean that the air pollution reached unhealthy levels in that particular point. In order to verify the capabilities of the system, a series of measurements have been taken, at the end of October 2019. The measurements were taken on a warm Saturday afternoon, with relatively low traffic, and still show the variation of air pollution in different locations.

**Figure 8:**
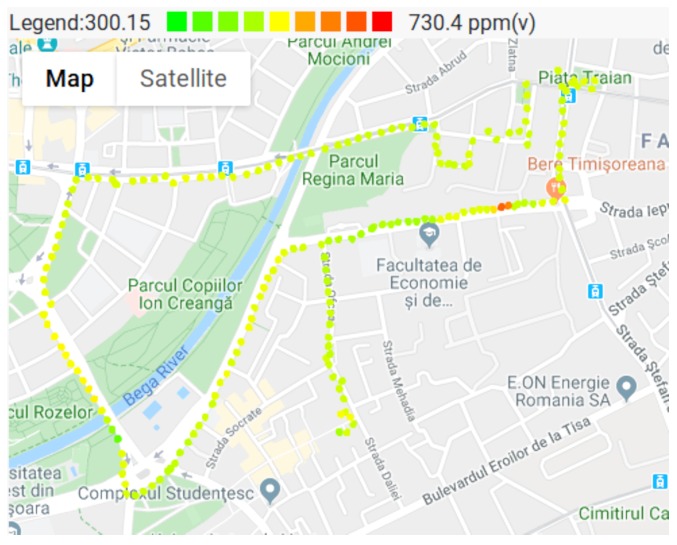
${\mathrm{CO}}_{2}$ measurements.

Figure 8, shows the ${\mathrm{CO}}_{2}$ pollution in various parts of the city. As the color codes show, most of the places in the city have normal concentrations, about 400 ppm. Higher concentrations (colored with orange and red) are measured in the eastern part of the map, where the ${\mathrm{CO}}_{2}$ reached 700 ppm, in a small area. Also on the West part of the map, there are some measurements around 500 ppm, due to the higher traffic on that specific street.

**Figure 9:**
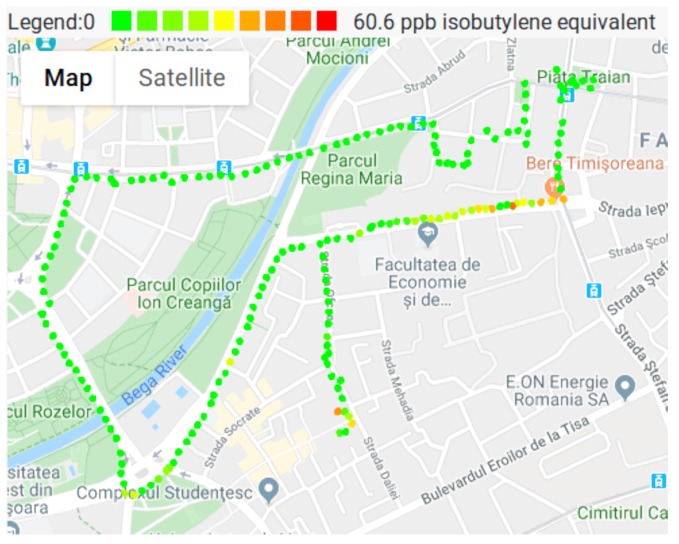
VOC measurements.

During the experiments, the VOC levels where also measured, the results being depicted in Figure 9. The minimum value measured by the VOC sensor is 0, and it is marked with green on the map. Most of the map is green, due to the low VOC pollution. It can be observed that VOC concentration reaches its maximum in the same location as the ${\mathrm{CO}}_{2}$, being approximately 55 ppb isobutylene equivalent. The source of the VOC pollution is relatively small, and as the sensor node departs form the pollution source, the VOC level drops significantly. On the Western part of the map, where ${\mathrm{CO}}_{2}$ concentrations where slightly above average, the VOC concentration remains zero.

The sensor node also measures environmental data, more exactly temperature and humidity. Each air quality sensor has an interval of temperature and humidity where it is able to measure. For example, the MIVS-VZ-89TE VOC sensor is working correctly if the temperature is between 0 °C and 50 °C. If the humidity and the temperature are outside of the bounds accepted by the sensor, the measured values are not correct, therefore they must be ignored.

**Figure 10:**
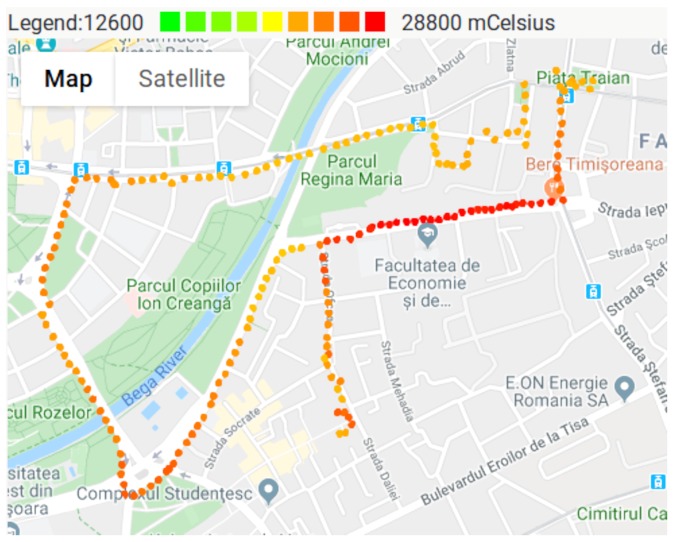
Temperature measurements.

Temperature values are mapped to Figure 10. During the measurements, the weather was unusually warm for that period, with temperatures of 22 °C in shadow, and 28 °C on the sun. For example, the South-western part of the map shows a sunny area.

**Figure 11:**
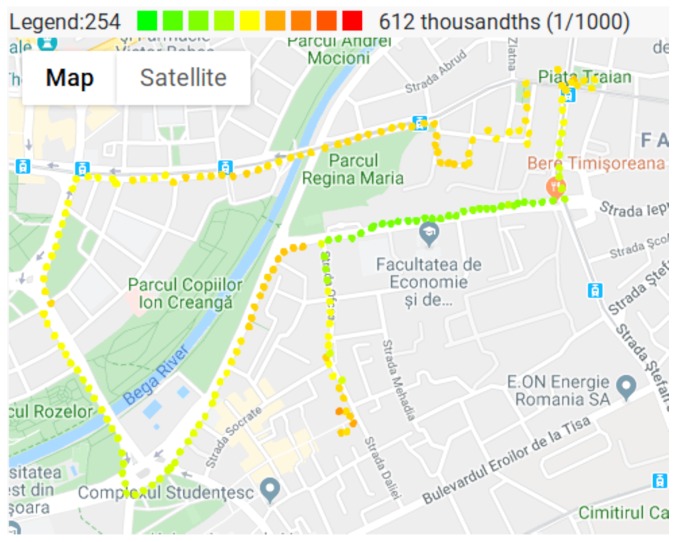
Humidity measurements.

Humidity values where also measured and can be visualized on Figure 11. The humidity values are also required in order to make sure that the sensors are in their operational range.

The maps presented in Figure 8, Figure 9, Figure 10 and Figure 11, show that pollutant concentrations can have high variation in space. The mobile sensor nodes are capable to capture the local variation in the pollutant concentrations. In the current case, it was able to identify a small zone with above average VOC and ${\mathrm{CO}}_{2}$ concentrations (western part of the map), and the slightly above average ${\mathrm{CO}}_{2}$ concentrations caused by a busy street. Therefore, the sensor nodes give a detailed image of the air pollution. Unlike fixed stations, they are able to determine the effects of a busy street to the neighborhood even in the level of meters. The limitation are given by the precision of the GPS.

## 4. Conclusions

In this work an air pollution measurement system based on a mobile multi-sensor module was proposed. The mobile sensor modules can upload measurements to a data server using mobile internet. Since such a connection may be insecure, data authenticity was preserved and user privacy was also addressed by means of proper cryptographic designs. To preserve anonymity, group signatures were applied to the measurements from the mobile multi-sensor module. These signatures were included in a blockchain-like structure together with the measurement data. This structure prevents tampering the measurements since any form of manipulation would be detected. A proof of concept version of the mobile multi-sensor module and the data servers was also implemented. Using this implementation, processing and transmission times were analyzed. Processing time is highly increased by the group signatures, which are done only rarely and moderately increased by the elliptic-curve cryptography algorithms for key-exchange. The transmission time is network dependent and optimizing it was out of scope of this paper. The power consumption of the system was also analyzed, in order to demonstrate that the proof-of-concept system is adequate for battery powered applications. Finally, pollution maps were created for both ${\mathrm{CO}}_{2}$ and VOC concentrations, which also capture the local variance of the pollutant concentrations.

## Figures and Tables

**Table 1:** System components.

Component	Role	Resources	Interfaces
Arduino Due	processing	AT91SAM3X8E microcontroller, 84 MHz, 96 Kb RAM	UART
Adafruit Fona 3G	network + GPS	SIM5320E chip	UART
Taoglas AP 25.E	GPS antenna	N/A	U.FL
ZL-PB350B2-PEX35B	GSM antenna	N/A	U.FL
External batteries	power source	N/A	N/A

**Table 2:** Used sensors.

Sensor	Role	Interfaces	Measurement Range	Precision	Price Range
T6713-6H	$CO_{2}$ Sensor	I2C	400–5000 ppm	±30ppm +3% of the reading	50–100 USD
MICS-VZ- 89TE	VOC Sensor	I2C	0–1000 ppb isobutylene equivalent	not specified	10–50 USD
DHT22	temperature and humidity sensor	I2C	−40–80 °C temperature 0–100 humidity	±0.2 °C temperature ±1 humidity	1–10 USD

**Table 3:** Cryptographic algorithms runtime analysis.

Algorithm	Input Size [byte]	Key Size [bit]	Runtime on AT91SAM3X8E [ms]	Runtime on Xeon E5-2676 v3 [ms]
SGS Signature	74	158	1877.39	7.22
SGS Verification	74	158	2873.67	11.36
ECDSA Signature	74	256	132.55	0.33
ECDSA Verification	74	256	141.08	0.33
ECDH Recover	-	256	126.24	0.30
ECDH Share	-	256	126.24	0.30
SHA256	320	-	0.43	0.003
HMAC (SHA256)	320	256	0.65	0.005
AES256 Encrypt	320	256	2.39	0.014
AES256 Decrypt	320	256	5.79	0.024

**Table 4:** Protocol processing time analysis.

Operation	Phase	Client Side Processing Time (ms)	Server Side Processing Time (ms)
Session key establishment	message 1	1.32	0.34
	message 2	470.39	0.33
	message 3	2263.69	12.43
Data upload	message 1	2.02	0.02
	message 2	0.95	0.07
Hash chain computation	not signed	0.25	0.003
	signed	2268.90	12.60

**Table 5:** Protocol processing time analysis.

Operation	Measured on	Processing Time (ms)	Transmission Delay (ms)	Total Time (ms)
Session key establishment	client side	2735.88	5717.54	8453.42
Session key establishment	server side	2747.18	5581.43	8328.62
Data upload with hash chain (not signed)	client side	3.31	5595.10	5598.41
Data upload with hash chain (signed)	client side	2271.97	5461.45	7733.41

## References

[1] Salvi C., Brickman S. Air Pollution Costs European Economies US$ 1.6 Trillion a Year in Diseases and Deaths. http://www.euro.who.int/en/media-centre/sections/press-releases/2015/04/air-pollution-costs-european-economies-us$-1.6-trillion-a-year-in-diseases-and-deaths,-new-who-study-says.

[2] Hien V.T.D., Lin C., Thanh V.C., Oanh N.T.K., Thanh B.X., Weng C.E., Yuan C.S., Rene E.R. (2019). An overview of the development of vertical sampling technologies for ambient volatile organic compounds (VOCs). J. Environ. Manag..

[3] Re G.L., Peri D., Vassallo S.D. (2014). Urban air quality monitoring using vehicular sensor networks. Advances onto the Internet of Things.

[4] Kumar P., Morawska L., Martani C., Biskos G., Neophytou M., Sabatino S.D., Bell M., Norford L., Britter R. (2015). The rise of low-cost sensing for managing air pollution in cities. Environ. Int..

[5] Postolache O., Pereira J., Girao P. (2009). Smart Sensors Network for Air Quality Monitoring Applications. IEEE Trans. Instrum. Meas..

[6] Lungu M., Stefu N. (2018). Study on particulate matter dispersion by correlating direct measurements with numerical simulations: Case study—Timisoara urban area. Int. J. Environ. Sci. Technol..

[7] Xiang Y. (2014). Mobile Sensor Network Design and Optimization for Air Quality Monitoring. Ph.D. Thesis.

[8] Xiang Y., Piedrahita R., Dick R.P., Hannigan M., Lv Q., Shang L. A Hybrid Sensor System for Indoor Air Quality Monitoring. Proceedings of the 2013 IEEE International Conference on Distributed Computing in Sensor Systems.

[9] Huang Q., Mao C., Chen Y. A Compact and Versatile Wireless Sensor Prototype for Affordable Intelligent Sensing and Monitoring in Smart Buildings. Proceedings of the ASCE International Workshop on Computing in Civil Engineering 2017.

[10] Lee U., Magistretti E., Gerla M., Bellavista P., Corradi A. (2009). Dissemination and Harvesting of Urban Data Using Vehicular Sensing Platforms. IEEE Trans. Veh. Technol..

[11] Hu S.C., Wang Y.C., Huang C.Y., Tseng Y.C. (2011). Measuring air quality in city areas by vehicular wireless sensor networks. J. Syst. Softw..

[12] Kim R., Lim H., Krishnamachari B. (2016). Prefetching-Based Data Dissemination in Vehicular Cloud Systems. IEEE Trans. Veh. Technol..

[13] Boubrima A., Bechkit W., Rivano H. (2017). Optimal WSN Deployment Models for Air Pollution Monitoring. IEEE Trans. Wirel. Commun..

[14] Wang Y.C., Chen G.W. (2017). Efficient Data Gathering and Estimation for Metropolitan Air Quality Monitoring by Using Vehicular Sensor Networks. IEEE Trans. Veh. Technol..

[15] Lai Y., Yang F., Su J., Zhou Q., Wang T., Zhang L., Xu Y. (2018). Fog-Based Two-Phase Event Monitoring and Data Gathering in Vehicular Sensor Networks. Sensors.

[16] Yim Y., Cho H., Kim S.H., Lee E., Gerla M. (2017). Vehicle location service scheme based on road map in Vehicular Sensor Networks. Comput. Netw..

[17] Nie W., Lee V.C.S., Niyato D., Duan Y., Liu K., Nutanong S. (2018). A Quality-Oriented Data Collection Scheme in Vehicular Sensor Networks. IEEE Trans. Veh. Technol..

[18] Devarakonda S., Sevusu P., Liu H., Liu R., Iftode L., Nath B. (2013). Real-time air quality monitoring through mobile sensing in metropolitan areas. Proceedings of the 2nd ACM SIGKDD International Workshop on Urban Computing.

[19] Liu D., Ning P., Li R. (2005). Establishing pairwise keys in distributed sensor networks. ACM Trans. Inf. Syst. Secur. (TISSEC).

[20] Liu A., Ning P. (2008). TinyECC: A configurable library for elliptic curve cryptography in wireless sensor networks. Proceedings of the 7th International Conference on Information Processing in SENSOR Networks.

[21] Koblitz N., Menezes A. (2005). Pairing-based cryptography at high security levels. Proceedings of the IMA International Conference on Cryptography and Coding.

[22] Zhang L., Wu Q., Solanas A., Domingo-Ferrer J. (2009). A scalable robust authentication protocol for secure vehicular communications. IEEE Trans. Veh. Technol..

[23] Wasef A., Shen X. (2010). Efficient group signature scheme supporting batch verification for securing vehicular networks. Proceedings of the 2010 IEEE International Conference on Communications.

[24] Zhu X., Jiang S., Wang L., Li H. (2013). Efficient privacy-preserving authentication for vehicular ad hoc networks. IEEE Trans. Veh. Technol..

[25] Wu L., Fan J., Xie Y., Wang J., Liu Q. (2017). Efficient location-based conditional privacy-preserving authentication scheme for vehicle ad hoc networks. Int. J. Distrib. Sens. Netw..

[26] Tangade S., Manvi S.S., Lorenz P. (2018). Decentralized and scalable privacy-preserving authentication scheme in VANETs. IEEE Trans. Veh. Technol..

[27] Boneh D., Boyen X., Shacham H. (2004). Short group signatures. Proceedings of the Annual International Cryptology Conference.

[28] Lo N.W., Tsai J.L. (2016). An Efficient Conditional Privacy-Preserving Authentication Scheme for Vehicular Sensor Networks Without Pairings. IEEE Trans. Intell. Transp. Syst..

[29] Asaar M.R., Salmasizadeh M., Susilo W., Majidi A. (2018). A Secure and Efficient Authentication Technique for Vehicular Ad-hoc Networks. IEEE Trans. Veh. Technol..

[30] Li C., Zhang X., Wang H., Li D. (2018). An Enhanced Secure Identity-Based Certificateless Public Key Authentication Scheme for Vehicular Sensor Networks. Sensors.

[31] Cui J., Xu W., Zhong H., Zhang J., Xu Y., Liu L. (2018). Privacy-Preserving Authentication Using a Double Pseudonym for Internet of Vehicles. Sensors.

[32] Javed M.A., Zeadally S., Hamid Z. (2018). Trust-based security adaptation mechanism for Vehicular Sensor Networks. Comput. Netw..

[33] Manvi S.S., Tangade S. (2017). A survey on authentication schemes in VANETs for secured communication. Veh. Commun..

[34] Gayathri N.B., Thumbur G., Reddy P.V., Muhammad Z.U.R. (2018). Efficient Pairing-Free Certificateless Authentication Scheme With Batch Verification for Vehicular Ad-Hoc Networks. IEEE Access.

[35] Dai F., Luo M., ZHANG Y. (2017). A Fault-Tolerant Batch Verification Scheme for Cloud Assisted VANETs. DEStech Trans. Eng. Technol. Res..

[36] Islam S.H., Obaidat M.S., Vijayakumar P., Abdulhay E., Li F., Reddy M.K.C. (2018). A robust and efficient password-based conditional privacy preserving authentication and group-key agreement protocol for VANETs. Future Gener. Comput. Syst..

[37] Liu Z., Xiong L., Peng T., Peng D.Y., Liang H.B. (2018). A Realistic Distributed Conditional Privacy- Preserving Authentication Scheme for Vehicular Ad Hoc Networks. IEEE Access.

[38] Ming Y., Shen X. (2018). PCPA: A Practical Certificateless Conditional Privacy Preserving Authentication Scheme for Vehicular Ad Hoc Networks. Sensors.

[39] Wan C., Zhang J. (2017). Efficient identity-based data transmission for VANET. J. Ambient. Intell. Humaniz. Comput..

[40] Ying B., Nayak A. (2017). Anonymous and Lightweight Authentication for Secure Vehicular Networks. IEEE Trans. Veh. Technol..

[41] CO_2_ Earth. https://www.co2.earth/.

[42] Törnqvist M., Ehrenberg L. (1994). On cancer risk estimation of urban air pollution. Environ. Health Perspect..

[43] Tsai W.T. (2016). Toxic Volatile Organic Compounds (VOCs) in the Atmospheric Environment: Regulatory Aspects and Monitoring in Japan and Korea. Environments.

[44] Kolumban-Antal G., Lasak V., Bogdan R., Groza B. Air pollution monitoring with secure low-cost Vehicular Sensor Networks. Proceedings of the 1st International Conference on Computational Methods and Applications in Engineering.

[45] Diffie W., Van Oorschot P.C., Wiener M.J. (1992). Authentication and authenticated key exchanges. Des. Codes Cryptogr..

[46] Unterluggauer T., Wenger E. (2014). Efficient pairings and ECC for embedded systems. Proceedings of the International Workshop on Cryptographic Hardware and Embedded Systems.

